# Hormonal Induction of Polo-Like Kinases (Plks) and Impact of Plk2 on Cell Cycle Progression in the Rat Ovary

**DOI:** 10.1371/journal.pone.0041844

**Published:** 2012-08-01

**Authors:** Feixue Li, Misung Jo, Thomas E. Curry, Jing Liu

**Affiliations:** 1 College of Life and Environmental Sciences, Hangzhou Normal University, Hangzhou, People’s Republic of China; 2 Department of Obstetrics and Gynecology, Chandler Medical Center, University of Kentucky, Lexington, Kentucky, United States of America; 3 College of Environmental and Resource Sciences, Zhejiang University, Hangzhou, People’s Republic of China; Clermont Université, France

## Abstract

The highly conserved polo-like kinases (Plks) are potent regulators of multiple functions in the cell cycle before and during mitotic cell division. We investigated the expression pattern of Plk genes and their potential role(s) in the rat ovary during the periovulatory period. Plk2 and Plk3 were highly induced both in intact ovaries and granulosa cells *in vivo* after treatment with the luteinizing hormone (LH) agonist, human chorionic gonadotropin (hCG). *In vitro,* hCG stimulated the expression of Plk2 in granulosa cells, but not Plk3. This induction of Plk2 expression was mimicked by both forskolin and phorbol 12 myristate 13-acetate (PMA). Moreover, Plk2 expression was reduced by inhibitors of prostaglandin synthesis or the EGF pathway, but not by progesterone receptor antagonist (RU486) treatment. At the promoter level, mutation of the Sp1 binding sequence abolished the transcriptional activity of the Plk2 gene. ChIP assays also revealed the interaction of endogenous Sp1 protein in the Plk2 promoter region. Functionally, the over-expression of Plk2 and Plk3 arrested granulosa cells at the G0/G1 phase of the cell cycle. In contrast, the knockdown of Plk2 expression in granulosa cells decreased the number of cells in the G0/G1 stage of the cell cycle, but increased granulosa cell viability. In summary, hCG induced Plk2 and Plk3 expression in the rat ovary. Prostaglandins and the EGF signaling pathway are involved in regulating Plk2 expression. The transcription factor Sp1 is important for Plk2 transcriptional up-regulation. Our findings suggest that the increase in Plk2 and Plk3 expression contributes to the cell cycle arrest of granulosa cells which is important for the luteinization of granulosa cells during the periovulatory period.

## Introduction

In females, an acute rise of luteinizing hormone (LH) released from the pituitary (called the LH surge) triggers ovulation and induces terminal differentiation of preovulatory granulosa cells to become luteal cells. The LH surge terminates granulosa cell proliferation and initiates a program of luteinization in which the cells stop their division and differentiate into luteal cells [1]. The cell cycle progression of periovulatory granulosa cells is controlled by a delicate balance between positive and negative regulators. Members of the family of polo-like kinases (Plks) were reported to be major cell cycle regulators in differentiated cells [2]. Plk1 was first reported to associate with mitotic spindle poles in the *Drosophila melanogaster* polo mutation [3]. So far five mammalian Plks family members have been characterized in murine and human, including Plk1 (Xenopus Plx1), Plk2/Snk (Xenopus Plx2), Plk3/Prk/FnK (Xenopus Plx3), Plk4/Sak and Plk5 [4].

Among the five members in the mammalian Plks family, Plk1 has been most thoroughly studied. The major function of Plk1 is promoting mitotic entry by phosphorylation of Cdc25C [5], [6]. In contrast to Plk1, Plk3 is an inhibitor of Cdc25C and G2/M transition and its over-expression results in rapid cell cycle arrest and apoptosis [7], [8]. The role of Plk2 in the cell cycle remains unclear. Plk2 is not required for cell division but seems to influence the G1 progression [9]. Burns et al. reported an anti-proliferative impact of Plk2 such that induction of Plk2 in a p53-dependent manner contributed to cell cycle arrest in the G2 phase and/or mitosis [10]. The fourth member, Plk4 is required for late mitotic progression and maintenance of chromosomal stability [11]. More recently, a new member of mammalian Plk family, Plk5, has been identified in murine and human cells [12], [13]. Due to lack of the kinase domain in human, Plk5 does not seem to have a role in cell cycle progression but retains important functions in neuron biology [12].

Considering the roles of Plk1-4 in regulating cytokinesis, we hypothesized that the LH surge induces these Plks and that their induction is involved in the transition of granulosa cells to luteal cells. In the present study, we investigated the periovulatory expression patterns of the Plk family members. We found that the *in vivo* expression of Plk2 and Plk3 was dramatically increased after treatment with hCG which was used to mimic the preovulatory LH surge. Moreover, hCG stimulated the expression of Plk2, but not Plk3, in granulosa cell cultures. Given the similar and dramatic induction of Plk2 by hCG both *in vivo* and *in vitro*, we further explored the regulatory mechanism of Plk2 expression and the potential role of Plk2 in periovulatory granulosa cells using an *in vitro* model.

## Results

### hCG induced the expression of Plks in periovulatory rat ovaries

The gonadotropin-primed immature female rat is a well accepted experimental model to examine ovulation as well as ovarian granulosa cell function [14], [15], [16]–[18]. Because LH and hCG share numerous functional homologies and act through the same receptor, hCG has been widely used as an effective substitute for LH to induce periovulatory processes. Therefore, in the present study, we used hCG to mimic the LH surge *in vivo* and the action of LH in preovulatory granulosa cell cultures.

**Figure 1:**
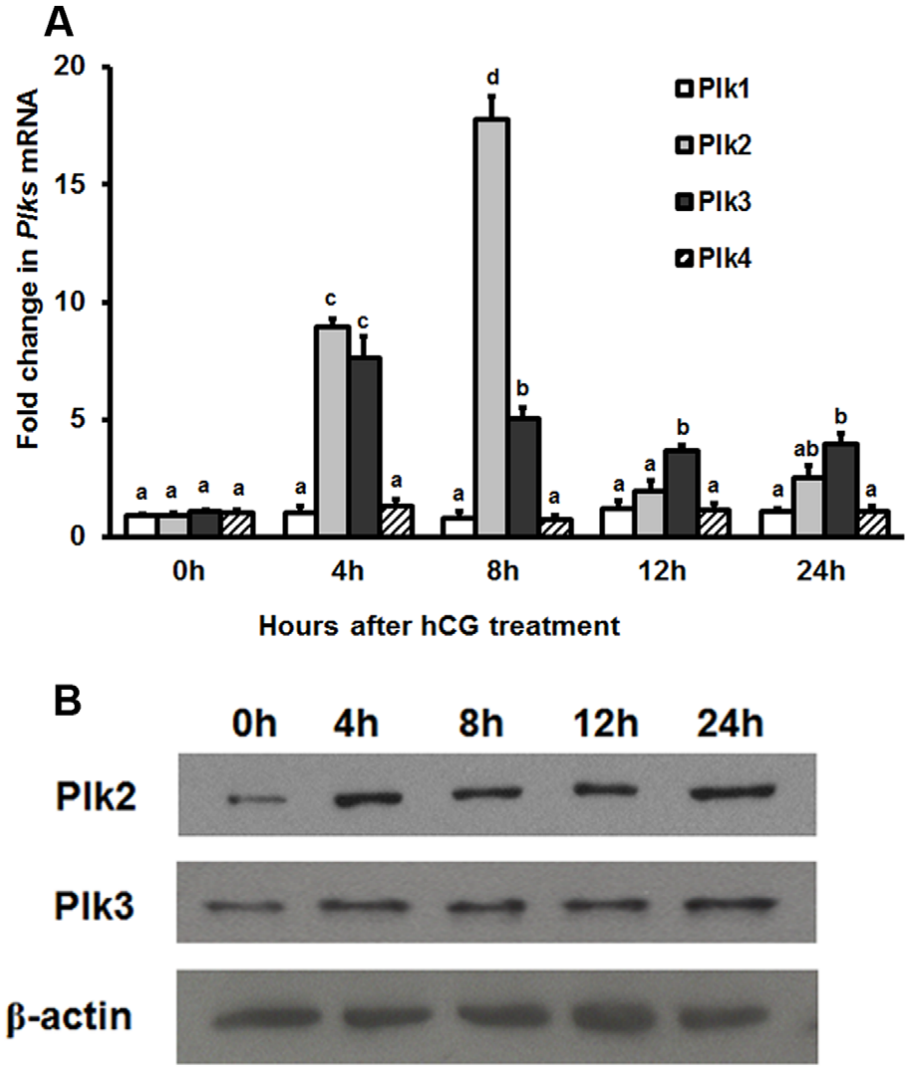
The expression of Plks in the rat ovary after hCG treatment. Rats were injected with PMSG for 48 h, treated with hCG, and ovaries collected at 0, 4, 8, 12, or 24 h after treatment. The data are expressed relative to the 0 h. (A) Real-time PCR was used to analyze the expression of Plk1, Plk2, Plk3 and Plk4 in intact preovulatory ovaries. Relative levels of mRNA for Plk1, Plk2, Plk3 and Plk4 were normalized to L32 in each sample (mean ± SEM; n = 3 independent experiments). (B) Plk2 and Plk3 protein levels in rat ovary were analyzed using western blot. Bars with no common superscripts are significantly different (*ρ* <0.05).

The expression patterns of Plk1, Plk2, Plk3 and Plk4 mRNA and protein were analyzed in ovaries collected at different times after hCG administration (Fig. 1). Both Plk2 and Plk3 gene expression increased after hCG although their expression pattern differed from each other. Plk2 mRNA expression increased between 4 and 8 h after hCG to the levels approximately 16-fold higher than that of the 0 h and returned to control levels by 12 h (Fig. 1A). Plk3 mRNA expression increased 7-fold at 4 h after hCG and remained high until 24 h (Fig. 1A). In contrast, the expression of Plk1 and Plk4 did not change after hCG treatment (Fig. 1A). Because Plk2 and Plk3, but not Plk1 and Plk4, are stimulated by hCG, we subsequently examined the protein levels of Plk2 and Plk3 *in vivo*. Both Plk2 and Plk3 protein levels increased at 4 h after hCG in the intact ovary (Fig. 1B).

### Effects of hCG on granulosa cell expression of Plk2 and Plk3 mRNA *in vivo* and *in vitro*

**Figure 2:**
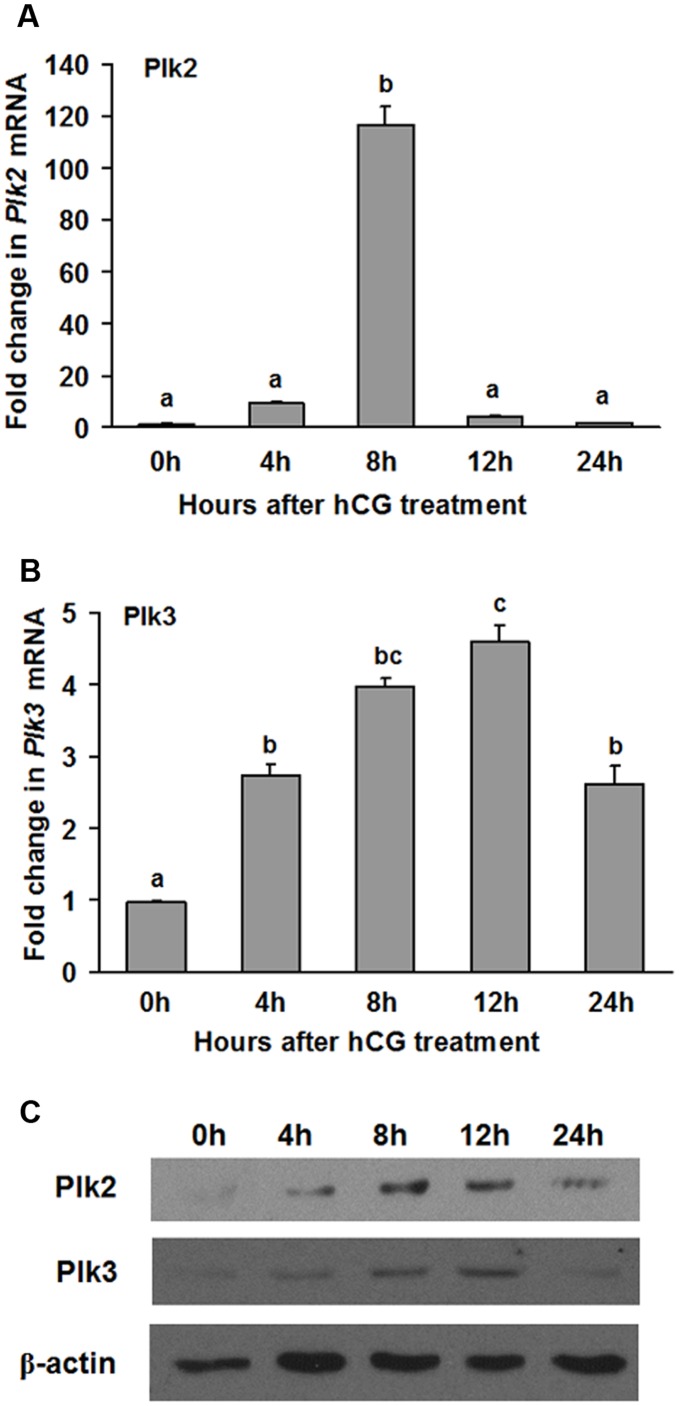
Plk2 and Plk3 expression increased in rat granulosa cells *in vivo* after hCG administration. Rat granulosa/luteal cells obtained from PMSG primed rat preovulatory ovaries were collected at 0, 4, 8, 12, or 24 h after hCG administration. (A-B) The expression of Plk2 (A) and Plk3 (B) mRNA was analyzed using real-time PCR. Relative levels of mRNA for Plk2 and Plk3 were normalized to L32 in each sample (mean ± SEM; n = 3 independent experiments). (C) Western blot shows Plk2 and Plk3 protein levels at different time points after hCG treatment. Bars with no common superscripts are significantly different (*ρ* <0.05).

Among the Plk family members, Plk2 and Plk3 expression prominently increased in the intact ovary. Therefore, their relative expression in the granulosa cell compartment was also assessed. Granulosa cells were isolated from ovaries at various times after hCG administration. The levels of Plk2 mRNA were highest at 8 h after hCG (Fig. 2A), similar to the peak levels observed in the whole ovary. Plk3 mRNA expression increased within 4 h after hCG and continued to increase until 12 h (Fig. 2B). Both Plk2 and Plk3 protein levels were increased at 4 h, reached peak levels at 8 h and 12 h, and declined at 24 h after hCG treatment (Fig. 2C).

**Figure 3:**
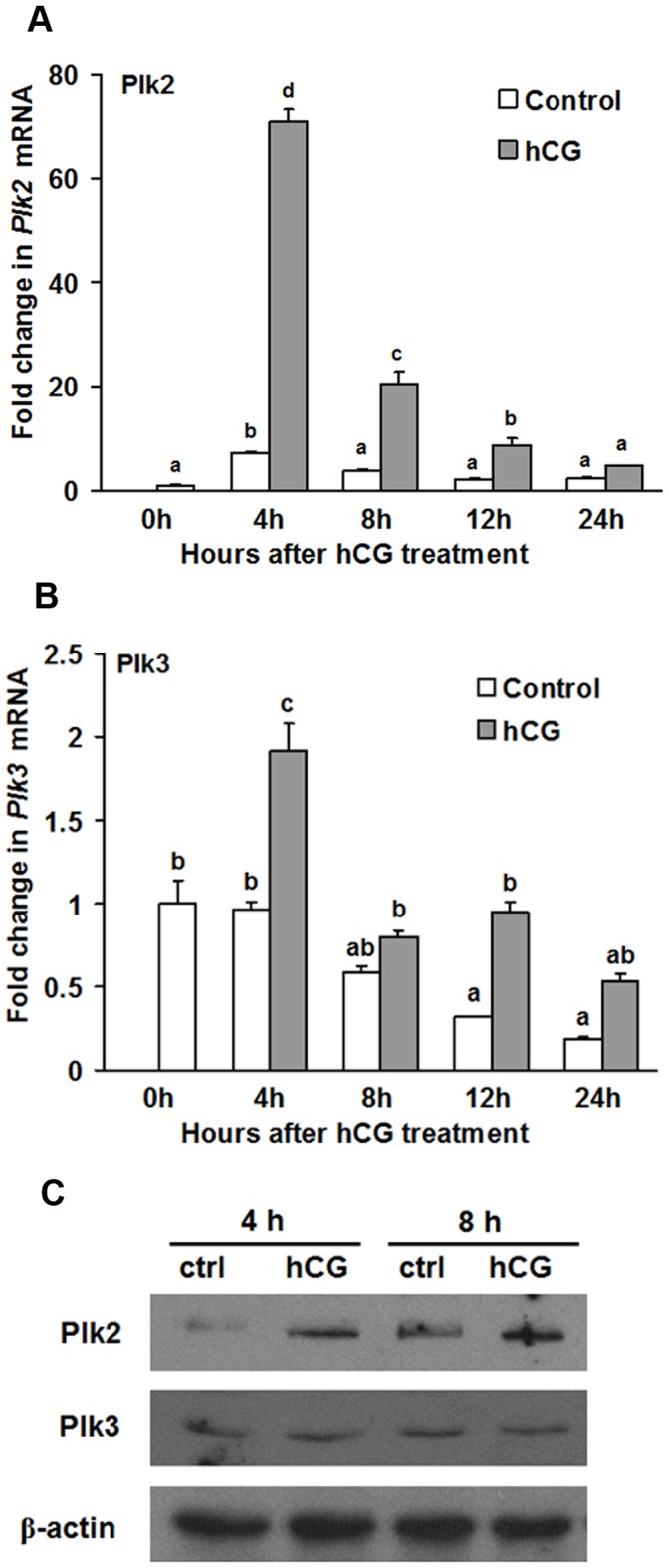
Stimulation of Plk2 and Plk3 expression by hCG in cultured rat granulosa cells *in vitro*. Granulosa cells obtained from rat preovulatory ovaries (48 h post-PMSG) were cultured in medium alone (Control) or with hCG (1 IU/ml) for different time points. (A-B) Real-time PCR analysis shows the expression of Plk2 (A) and Plk3 (B) mRNA. Relative levels of Plk2 and Plk3 mRNA were normalized to L32 in each sample (mean ± SEM; n = 3 independent culture experiments). (C) Western blot shows Plk2 and Plk3 protein levels at 4 and 8 h after hCG treatment in rat granulosa cells *in vitro*. Bars with no common superscripts are significantly different (*ρ* <0.05).

We further determined whether the induction of Plk2 and Plk3 in granulosa cells *in vivo* can be mimicked *in vitro* by the hCG treatment. The results showed that hCG induced a similar pattern of Plk2 mRNA expression in cultured granulosa cells (Fig. 3A) comparable to its expression *in vivo* (Fig. 2A), with the exception that the highest expression was observed at 4 h after hCG *in vitro*. The increase of Plk2 protein was observed at 4 h and 8 h after hCG treatment (Fig. 3C). Interestingly, the Plk3 expression pattern in cultured granulosa cells was different from its *in vivo* expression pattern. The expression of Plk3 mRNA increased 2-fold at 4 h by hCG treatment compared to control (Fig. 3B), but quickly declined to the 0 h value by 8 h in the culture. Moreover, Plk3 protein levels were not increased by hCG (Fig. 3C).

### Regulation of Plk2 mRNA expression

**Figure 4:**
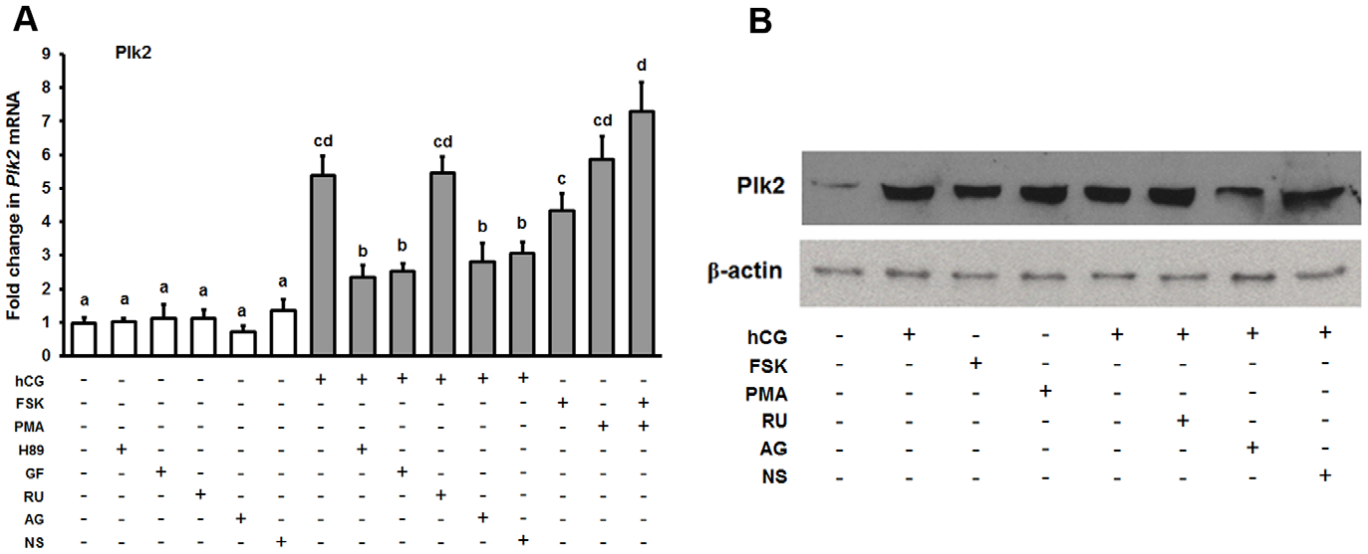
Regulation of Plk2 expression in granulosa cells *in vitro*. (A) Real-time PCR analysis shows the expression of Plk2 in rat granulosa cells cultured in medium alone (Ctrl) or with hCG (1 IU/ml), forskolin (FSK, 10 μM), phorbol 12 myristate 13-acetate (PMA, 20 nM) or FSK+PMA for 4 h. The expression of Plk2 was also analyzed in granulosa cells cultured with hCG in the absence or presence of the inhibitor of PKA (H89, 10 μM), inhibitor of PKC (GF109203X, 1 μM), EGF receptor tyrosine kinase selective inhibitor (AG1478, 1μM), the PGR antagonist (RU486, 1μM), or the PTGS2 inhibitor (NS-398, 1μM) for 4 h. Relative levels of mRNA for Plk2 were normalized to L32 in each sample (mean ± SEM; n = 3 independent culture experiments). (B) Western blot shows Plk2 protein levels in rat granulosa cells after different treatments. GF, GF109203X; AG, AG1478; RU, RU486; NS, NS-398. Bars with no common superscripts are significantly different (*ρ* <0.05).

The fact that Plk2 expression was dramatically increased by hCG suggests that it may play an important role in periovulatory granulosa cells. Thus, we further investigated the regulation of Plk2 expression. It is well known that LH/hCG activates both protein kinase A (PKA) and protein kinase C (PKC) signaling pathways in preovulatory granulosa cells [19]. To determine which signaling pathway(s) is involved in the up-regulation of Plk2 mRNA expression in response to hCG stimulation, granulosa cells from rat preovulatory ovaries (48 h post-PMSG) were cultured with hCG, forskolin (FSK) which is an activator of adenylate cyclase, or an activator of PKC, phorbol 12 myristate 13-acetate (PMA) for 4 h. The treatments of FSK, PMA, or FSK+ PMA increased Plk2 mRNA and protein levels in preovulatory granulosa cell cultures (Fig 4A and B). Both PKA and PKC pathway inhibitors (H89 and GF109203X) could block hCG-dependent Plk2 mRNA induction at 4 h (Fig. 4A). These results suggested that both PKA and PKC signaling pathways are involved in hCG-induced Plk2 expression.

hCG also sets in motion a number of steps which are crucial for follicular rupture and oocyte release including activation of epidermal growth factor (EGF) signaling and induction of progesterone receptors (PGR) and prostaglandin-endoperoxide synthase 2 (PTGS2) [20]. We tested whether the up-regulation of Plk2 mRNA is mediated by the hCG-induced activation of these signaling pathways using RU486 to block PGR action, NS398 to inhibit PTGS2, and AG1478 to prevent EGF signaling. AG1478 and NS398 reduced the hCG-stimulated expression of Plk2 by hCG (Fig. 4A and B), whereas RU486 treatment had no effect on Plk2 mRNA induction (Fig. 4A and B).

### Reduction of Plk2 promoter activity by mutation of Sp1 binding sequences in preovulatory granulosa cells

**Figure 5:**
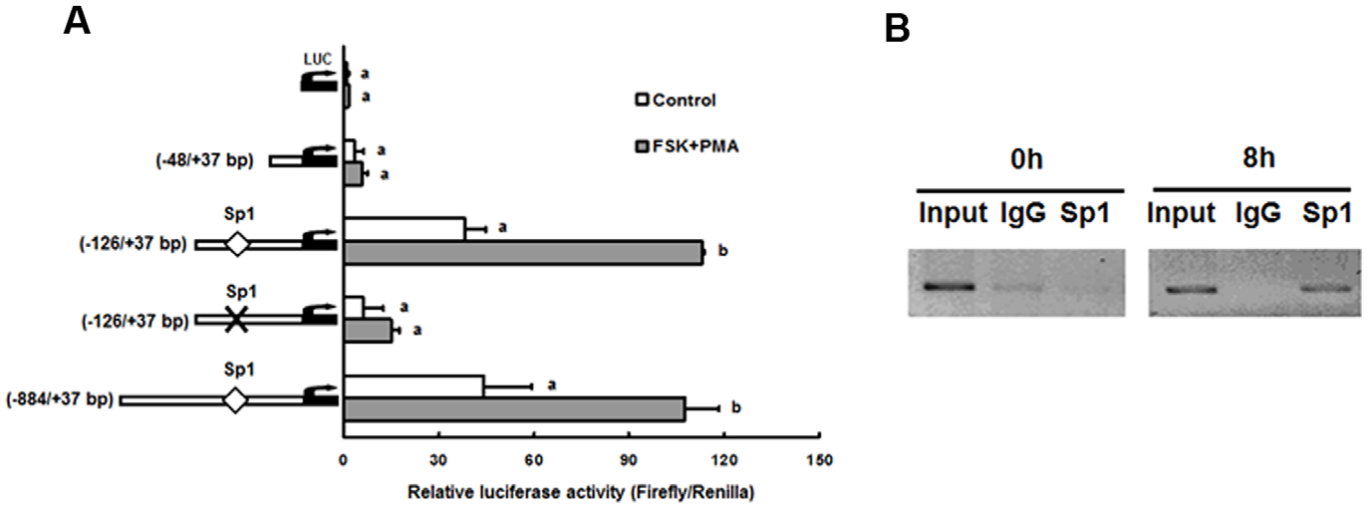
Sp1 binds to the Plk2 promoter and regulates the transcriptional activity of the Plk2 promoter reporter construct. (A) Plk2 promoter reporter constructs (−884/+37, −126/+37, and −48/+37 bp) or empty luciferase reporter vector (LUC) were transfected into granulosa cells isolated from PMSG-primed immature rats. Granulosa cells were then treated with or without FSK (10 μM) +PMA (20 nM) for 8 h. Firefly luciferase activities were normalized to Renilla luciferase activities. (B) ChIP detection of Sp1 transcription factor binding to the rat Plk2 promoter region in granulosa cells. A DNA fragment (−129 to +31 bp) containing the Sp1 binding site was enriched in chromatin samples immunoprecipitated with Sp1 antibody. Each experiment was performed in triplicate and the experiment was repeated at least three times. Bars with no common superscripts are significantly different (*ρ* <0.05).

To investigate which transcription factors are important for Plk2 transcription, three Plk2 promoter reporter constructs (−884/+37, −126/+37, and −48/+37 bp) were transfected into granulosa cells isolated from PMSG-primed immature rats. The transfected granulosa cells were treated with FSK+PMA which mimics the action of an ovulatory dose of LH/hCG [15], [16]–[18]. Luciferase activity of −884/+37 and −126/+37 bp Plk2 construct was stimulated by FSK+PMA, whereas FSK+PMA did not stimulate luciferase activity of the −48/+37 bp construct (Fig. 5A). This observation suggested that the -126 bp/−48 bp region was important for FSK+PMA-stimulated Plk2 promoter activity. In granulosa cells, Sp1 was found to be activated to promote the transcription of genes involved in ovulation or luteinization after an hCG stimulus [21]. As there is one Sp1 binding site in the −126 bp/−48 bp region of the rat Plk2 promoter, we determined whether Plk2 mRNA expression is regulated by the Sp1 transcription factor. Mutation of the Sp1 binding site in −126/+37 bp Plk2 promoter construct resulted in reduced agonist-stimulated transcription activity, suggesting that Sp1 is involved in Plk2 transcriptional regulation (Fig. 5A). The mutation of Sp1 also decreased the basal activity of Plk2 promoter construct, suggesting that Sp1 is important for Plk2 basal transcription (Fig. 5A).

### Endogenous Sp1 binds to the Plk2 promoter region in periovulatory granulosa cells

To further determine the interaction between Sp1 protein and Sp1 binding motifs in the Plk2 promoter *in vivo*, ChIP assays were performed on chromatin samples extracted from periovulatory granulosa cells. PCR analysis revealed that immunoprecipitation of endogenous Sp1 enriched chromatin fragments containing the Sp1 binding sequence in the promoter region compared to that of normal rabbit IgG at 8 h post-hCG (Fig. 5B). This result indicated that endogenous Sp1 protein was associated with the Plk2 promoter in periovulatory granulosa cells.

### Cell cycle analysis of granulosa cells after over-expression or knockdown of Plk2

**Figure 6:**
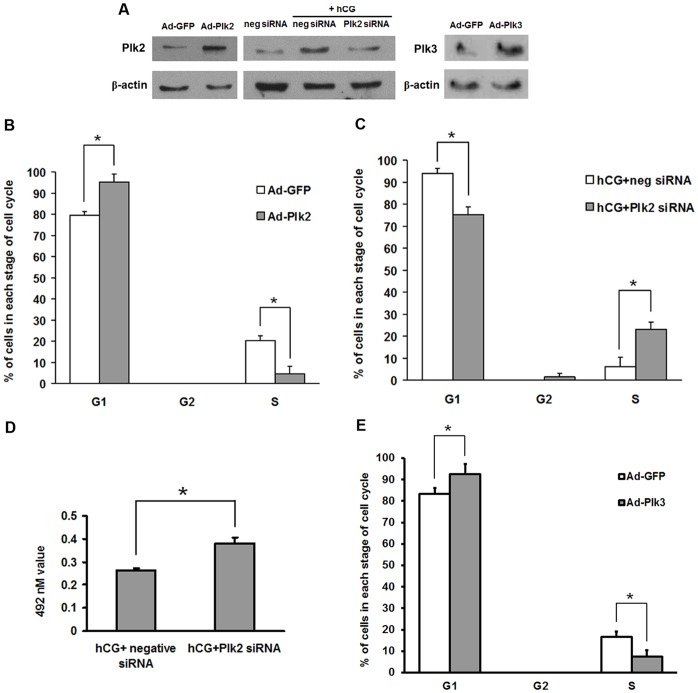
Cell cycle analysis of granulosa cells after over-expression of rat Plk2 and Plk3 or knockdown of rat Plk2. (A) Western blot shows that Plk2 is either highly expressed or silenced in granulosa cells which were treated with Ad-Plk2 adenovirus or Plk2 siRNA, respectively. Plk3 is highly expressed in granulosa cells which were treated with Ad-Plk3 adenovirus. (B) Granulosa cells infected with the Ad-Plk2 or Ad-GFP (control) adenovirus vector for 48 h were analyzed for cell cycle kinetics. (C) Granulosa cells treated with Plk2 siRNA or scrambled siRNA (control) for 48 h were analyzed for cell cycle kinetics. The cells at each stage of cell cycle, G1, S, and G2 are depicted. (D) Granulosa cells were treated with Plk2 siRNA or negative siRNA (control) in the presence of hCG for 24 h. Granulosa cell viability was measured using the MTS assay. (E) Granulosa cells infected with the Ad-Plk3 or Ad-GFP (control) adenovirus vector for 48 h were analyzed for cell cycle kinetics. The experiments were repeated at least three times and analyzed by paired t tests. *, ρ <0.05.

To investigate the potential function of Plk2 in granulosa cells, we used over-expression or siRNA approaches. In the over-expression experiment, granulosa cells from preovulatory ovaries were infected with either a recombinant Ad-GFP or Ad-Plk2 adenovirus. Western blot results confirmed that Plk2 was highly expressed in the granulosa cells (Fig. 6A). The over-expression of Plk2 resulted in an alteration in the population of granulosa cells entering the cell cycle (Fig. 6B). The percentage of cells in the G0/G1 phase increased from 79.6% to 95.4% in granulosa cells over-expressing Plk2, whereas the percentage of cells in the S phase markedly decreased from 20.4% to 4.6% (Fig. 6B). In the siRNA experiment, Plk2 protein knockdown was observed in granulosa cells transfected with Plk2 siRNA in the presence of hCG treatment (Fig. 6A). In contrast to the data from the over-expression study, Plk2 siRNA reduced the percentage of cells in the G0/G1 phase from 93.9% to 75.4%, increased the percentage of cells in the G2 phase from 0 to 1.5% and S phase from 6.1% to 23.1% (Fig. 6C). An MTS assay was also used to determine the effect of Plk2 on the viability of granulosa cells. The results of MTS assay showed that the viability of granulosa cells increased significantly after Plk2 siRNA treatment (Fig. 6D), which suggested that the number of viable granulosa cells increased after Plk2 siRNA treatment. These findings suggest that Plk2 expression blocks granulosa cell proliferation.

### Cell cycle analysis of granulosa cells after over-expression of Plk3

To investigate the effects of Plk3 on cytokinesis of granulosa cells, Plk3 over-expression adenovirus vector Ad-Plk3 was also constructed. The over-expression of Plk3 resulted in a similar alteration in the population of granulosa cells entering the cell cycle as observed for overexpression of Plk2 (Fig. 6E). The percentage of cells in the G0/G1 phase increased from 83.3% to 92.6% after over-expressing of Plk3, whereas the percentage of cells in the S phase markedly decreased from 16.7% to 7.4% (Fig. 6E).

## Discussion

In response to the LH surge, proliferating granulosa cells undergo an irreversible exit from the cell cycle and differentiate to luteal cells. Numerous signals and regulators have been reported to be involved in the process. In this study, we demonstrated that both Plk2 and Plk3 expression are induced prior to ovulation and that manipulation of Plk2 or Plk3 expression impacts the cell cycle kinetics of rat granulosa cells.

There are limited reports on the expression and localization of the mammalian Plk family in the ovary. Plk1 mRNA was detected in mouse ovarian tissue [22], and it was detected in the mouse oocyte where it was required for oocyte meiotic maturation [23]. Gene screening of ovaries from gonadotropin-primed immature rats revealed that Plk2 was highly induced by hCG [24]. In situ hybridization analysis showed that Plk2 was localized to granulosa cells of preovulatory follicles [24]. In the surveys of various tissues, relatively high levels of Plk3 mRNA were detected in the mouse and human ovary [25], [26]. The mRNA expression for Plk4 was almost undetectable in the mouse and human ovarian tissues [27], [28]. In the present study, mRNA expression for Plk1 and Plk4 did not change in periovulatory ovaries or isolated granulosa cells in response to hCG, suggesting that they may not be involved in the transition of granulosa to luteal cells.

The expression of Plk2 and Plk3 was highly induced in granulosa cells *in vivo* after hCG treatment. The induction of Plk2 mRNA in granulosa cells *in vivo* is much higher than that in whole ovary suggesting that Plk2 is predominately expressed in the granulosa cell compartment. However, previous reports have also demonstrated Plk2 in the theca interstitial cells [29]. For example, *in situ* hybridization revealed that there is substantial signal in the theca interstitial cells at 1 and 3 h after hCG [29]. In contrast to Plk2 mRNA expression, the induction of Plk3 mRNA expression was similar between the granulosa cell compartment and intact ovary implying that other ovarian compartments contribute to the overall Plk3 mRNA expression. Interestingly, the expression profiles of Plk3 differ between *in vitro* and *in vivo*. We would propose that this difference in response to hCG relates to the isolation procedure of the rat granulosa cells including a loss of cell-cell contact, a disruption of the cell matrix interaction, and/or a loss of communication with the thecal cell compartment. The up-regulation of Plk2 and Plk3 after hCG led us to hypothesize that they may be involved in the process of granulosa cell luteinization.

Both the PKA and PKC signaling pathways are known to be activated by LH/hCG in preovulatory granulosa cells [19]. In the present study, both PKA and PKC pathways appear to be involved in the regulation of Plk2 as either FSK or PMA can mimic hCG induction of the Plk2 expression. In the ovary, the LH surge stimulates a number of steps that include the activation of progesterone, prostaglandin, and the EGF signaling pathways [20]. The expression pattern of PTGS2, PGR, and EGF-related peptides proceeds or is parallel to that of Plk2 expression. Various specific inhibitors were used to test whether the hCG-stimulated up-regulation of Plk2 is mediated by the action of progesterone, prostaglandins, or EGF-related peptides. The results indicate that prostaglandins and the EGF signaling pathway, but not progesterone, play important roles in regulating hCG-dependent Plk2 expression.

Sp1 as a transcription factor has been reported to enhance or repress transcription of genes involved in differentiation, cell cycle progression, and oncogenesis [30]. The expression of Sp1 is constitutive in granulosa cells [31]. It is well recognized that Sp1 is activated to promote the transcription of target genes which are involved in ovulation and luteinization after the LH surge [21]. These genes include serum/glucocorticoid inducible-protein kinase (Sgk) [32], early growth response protein-1 (Egr-1) [33], cholesterol side-chain cleavage cytochrome P450 (CYP11A; P450scc) [34], and progesterone receptor [30]. As one Sp1 binding site was found in rat Plk2 promoter, we determined whether Plk2 mRNA transcription is also regulated by Sp1. The mutation of the Sp1 binding site on the Plk2 promoter markedly reduced the basal and FSK+PMA-induced transcriptional activity in granulosa cells. The direct binding of Sp1 to the Plk2 promoter was also observed *in vivo* by ChIP analysis. These findings suggest that Sp1 is important for hCG mediated Plk2 transcriptional induction as well as its basal transcription.

In response to the LH surge granulosa cells of preovulatory follicles stop proliferation and begin to differentiate into luteal cells. Many cell cycle regulators have been reported to play roles in the process of luteinization, such as p27 ^Kip1^, CyclinD2 and B cell translocation gene (BTG) [16], [35], [36]. In the present study, in the periovulatory granulosa cells undergoing differentiation to luteal cells, LH/hCG dramatically induced the expression of Plk2 and Plk3, which are supposed to be negative regulators of the cell cycle. In mice embryos lacking Plk2, Plk2 was not required for cell division but seemed to influence the G1 progression of embryonic fibroblasts [9]. Plk2 deficient tumors grew much larger than control tumors suggesting a tumor suppressor function for Plk2 [37]. The loss of Plk2 as a result of methylation dependent silencing of the Plk2 gene has been discovered in patients with Burkitt’s lymphoma [38]. These observations indicate an anti-proliferative impact of Plk2 during mitosis. It has been shown that Plk3 regulated M phase functions through direct regulation of Cdc25C and over-expression of active Plk3 induced apparent cell cycle G2/M arrest followed by apoptosis [7], [8]. Thus, we further investigated the effect of Plk2 and Plk3 on granulosa cell proliferation. Over-expression of Plk2 and Plk3 in granulosa cells resulted in a significant increase in the number of cells in the G0/G1 stage of the cell cycle. In contrast, silencing Plk2 expression in granulosa cells decreased the number of cells in the G0/G1 stage of the cell cycle. In the mean time, the viability of granulosa cells increased significantly after Plk2 siRNA treatment which may be due to the increase of proliferation. Our results suggest that both Plk2 and Plk3 contribute to the cell cycle arrest of granulosa cells after LH surge.

In summary, the present study demonstrates that both Plk2 and Plk3 are highly expressed in granulosa cells *in vivo* after hCG administration, but only Plk2 is induced by hCG *in vitro*. The induction of Plk2 is mediated by the PKA and PKC pathways, as well as the hCG-induced activation of EGF signaling pathway and prostaglandins. The transcription factor Sp1 is important for Plk2 transcription. Our novel findings suggest Plk2 and Plk3 may regulate the cell cycle arrest of granulosa cells after the LH surge which is critical for granulosa cell luteinization.

## Materials and Methods

### Ethics statement

The experimental protocol was approved by the University of Kentucky Institutional Animal Care and Use Committee and Institutional Animal Care and Use Committee at Zhejiang University and complied with the principles of laboratory animal care.

### Materials and reagents

Molecular biological enzymes and molecular size markers were purchased from Toyobo (Osaka, Japan). Trizol™ and pCRII-TOPO Vector were purchased from Invitrogen™ Life Technologies, Inc. (Carlsbad, CA). Chemicals and reagents (PMSG, hCG, FSK, PMA, H89, GF109203X, RU486, NS398, and AG1478) were all purchased from Sigma Chemical Co. (St. Louis, MO) unless mentioned.

### Tissue collection

Immature female Sprague-Dawley rats were provided by Harlan, Inc.(Indianapolis, IN) and Zhejiang University Laboratory Animal Center and maintained on a 12L:12D cycle as described previously [39], [40]. On the morning of day 22–23 rats were injected with pregnant mare’s serum gonadotropin (PMSG, 10 IU) s.c to stimulate ovarian follicle development. Forty eight hours later animals were injected with hCG (5 IU) s.c. to induce ovulation. Ovaries were collected at 0 h (i.e. time of hCG administration) or defined times after hCG administration (n = 3–5 animals/time point). Ovaries were stored at −80°C for later isolation of total RNA or protein.

### Isolation and culture of rat granulosa cells

Ovaries were collected 48 h after PMSG administration and processed as described previously [39], [40]. The cells were pooled, filtered, pelleted by centrifugation at 200× g for 5 min, and resuspended in HyQ MEM-RS (ThermoFisher Scientific, Waltham, MA, USA) media supplemented with 0.05 mg/ml gentamicin and 1× insulin, transferrin, and selenium. The granulosa cells were seeded in 10cm plates at 1×10^6^/well and cultured at 37°C in a humidified atmosphere of 5% CO_2_. The cells were treated with various reagents (hCG, FSK, PMA, H89, GF109203X, RU486, NS398, and AG1478) for time points outlined below for each experiment. Each experiment was performed at least three times.

### Quantification of mRNA for Plk genes

Real-time PCR was used to measure expression of Plk1, Plk2, Plk3 and Plk4 mRNA *in vitro* and *in vivo*. Oligonucleotide primers for rat L32 (Forward 5′-GAA GCC CAA GAT CGT CAA AA- 3′, Reverse 5′-AGG ATC TGG CCC TTG AAT CT -3′ ), rat Plk1 (Forward 5′-CCT ATT ACC TGC CTC ACC ATC C-3′, Reverse 5′-CCT CAT TTG TCT CCC GAA CC-3′ ), rat Plk2 (Forward 5′- GAA TCC TGC ACC ATA AGC-3′, Reverse 5′-TCT GCC TGA GGT AGT ATC G-3′), rat Plk3 (Forward 5′-GAT AAC ATG GAA CTG AAG G-3′, Reverse 5′- TAC ATG ACA CAA CCA AGG-3′ ) and rat Plk4 (Forward 5′- AAG CAT CTC TTC AAG TCT TCC-3′, Reverse 5′-CTC CAT ACC TAG TTG TCT GAC C-3′ ) were designed using OMIGA 2.0 software (Oxford Molecular Ltd, Madison, WI, USA) and synthesized by Shanghai Sangon Biological Engineering Technology & Services (Shanghai, China). The specificity for each primer set was confirmed by electrophoresis of the PCR products on a 2.0% agarose gel. The PCR products were sequenced before using. The melting (dissociation) curve was analyzed using a 7300 Real-Time PCR System (Applied Biosystems, Foster City, CA, USA) after each real-time PCR. The real-time PCRs were carried out as previously described [41], [42]. The relative amount of each Plk gene transcript was calculated using the 2^−ΔΔCT^ method and normalized to the endogenous reference gene L32.

### Western blot analysis

Western blotting was performed as described previously [16], [17]. Total protein was isolated from intact ovarian tissues and granulosa cells. Thirty microgams of protein was separated on a 10% SDS-PAGE gel and transferred to a nitrocellulose membrane (Whatman, Sanford, ME). Blots were incubated with the primary antibodies for Plk2 (1∶200, Sigma-Aldrich, St Louis, MO), Plk3 (1∶100, Sigma-Aldrich) or actin (1∶2000, Cell Signaling Technology, Danvers, MA) overnight at 4°C. Blots were analyzed using an enhanced chemiluminescence detection system (Pierce, Rockford, IL) and exposed to x-ray film.

### Generation of rat Plk2 promoter constructs and granulosa cell transfection

Genomic DNA was isolated from rat tail samples using an easy-DNA kit (Invitrogen). 921-bp (−884/+37), 163-bp (−126/+37) and 85-bp (−48/+37) fragment of the Plk2 gene were amplified using the primers attached with restriction enzyme sites (KpnI and NheI). Fragments of Plk2 promoter were cloned into the pGL3 basic vector (Promega, Madison, WI). Site-directed point mutations of the Plk2 promoter were generated using a QuickChange II site-directed mutagenesis kit according to the manufacturer’s protocol (Stratagene). The sequences of the oligonucleotide primers used to generate Plk2 promoter containing mutation (shown in lowercase) are following: mutant (5′- TGA CGT CAC GAG GCC CaattC CAC CCA GCA GGC GCG -3′).

Granulosa cells isolated from immature rats (48 h after PMSG) were seeded into 96 well plates at 1×10^5^/well. Granulosa cells were transfected with respective firefly luciferase reporter plasmids (pGL3-basic vector or pGL3-Plk2 promoter constructs, 0.2µg/well) and Renilla luciferase vector (pRL-TK vector) using a Lipofectamine 2000 reagent (Invitrogen). The next day, cells were treated with forskolin (FSK; 10 μM) and phorbol 12-myristate 13-acetate (PMA; 20 nM) for 4 h. The cells were then harvested to measure firefly and Renilla luciferase activities using a dual-luciferase reporter assay system (Promega). Firefly luciferase activities were normalized by Renilla luciferase activities and each experiment was performed in triplicate at least three times.

### Chromatin immunoprecipitation (ChIP) analysis

ChIP assay was performed on Sp1 in the Plk2 promoter region using a ChIP kit (Upstate Biotechnology, Inc., Lake Placid, NY) as described previously [18]. Granulosa cells were collected from PMSG-primed immature rat ovaries at 0 and 8 h after hCG injection. The nuclei of cells were released in lysis buffer and sonicated to obtain DNA fragments (approximately 100–500bp). Chromatin was immunoprecipitated overnight at 4°C with Sp1 antibody (5 µg/reaction; Abcam) or normal rabbit IgG (5 µg/reaction; Santa Cruz Biotechnology) as a negative control. The input chromatin (1∶10 dilution) and immunoprecipitated chromatin were analyzed by PCR. The primers were designed to amplify fragments spanning the Sp1 motif in the Plk2 promoter, forward: 5′-ACC CCG CAT CTA TCC ACA GTG C-3′, reverse: 5′- CTC CCT ACT CTC TAG TCC GAC G-3′. PCR products were run on a 2% agarose gel. Experiments were performed at least three times.

### Generation of recombinant adenovirus vector and granulosa cell infection

The AdEasy XL adenoviral vector system kit (Stratagene) was used to construct Plk2 and Plk3 recombinant adenovirus. The processes for generating and propagating recombinant adenoviruses were described previously [17]. Briefly, the full length of rat Plk2 and Plk3 genes were cloned into the pShuttle-CMV vector and then a recombinant Ad-Plk2 and Ad-Plk3 plasmids were generated by homologous recombination. Ad-Plk2 and Ad-Plk3 plasmids were transfected into AD-293 cells (Stratagene) where viral particles were further amplified. Adenoviruses were collected and titered using the AdEasy viral titer kit (Stratagene).

Rat granulosa cells collected at 48 h after PMSG priming were cultured in HyQ MEM-RS medium for 4 h before addition of the adenoviruses. The granulosa cells were exposed to Ad-Plk2, Ad-Plk3 or Ad-GFP at a multiplicity of infection (MOI) of 50 pfu/cell for 2 h. Then the medium was replaced with fresh HyQ MEM-RS medium. Approximately a 70% infection efficiency of GFP-adenovirus in granulose cells was routinely observed. Granulosa cells were collected for total RNA isolation, protein extraction, or flow cytometric analysis after adenovirus exposure for 48 h.

### Knockdown of Plk2 by small interfering RNA (siRNA) in granulosa cells *in vitro*

Granulosa cells were collected from immature rats 48 h after PMSG administration. Cells were transfected with the specific siRNA against Plk2 (Invitrogen, Carlsbad, CA) or scrambled siRNA (Invitrogen) using the Lipofectamine 2000 reagent (Invitrogen) according to the manufacturer’s instructions. Four hours later, transfection media were replaced with fresh culture media and the cells were treated with hCG (1 IU/ml) for further 48 h. The cells were collected for flow cytometric analysis or processed to prepare cell lysates for Western blot analyses.

### Flow cytometric analysis of granulosa cells

To determine the impact of Plk2 and Plk3 on cell cycle kinetics, granulosa cells treated with Ad-Plk2 and Ad-Plk3 adenovirus or Plk2 siRNA as described above. Granulosa cells were suspended using 3% trypsin after culture and then stained for DNA content. Briefly, ribonuclease A was added to cells (1×10^6^) and incubated at 37°C for 30 min. Then, granulosa cells were resuspended in propidium iodide (50 µg/mL) and incubated for 15 min in the dark at 4°C. A FACS Calibur flow cytometer (Becton Dickinson, Franklin Lakes, NJ) at Zhejiang University was used to determine the cell cycle distribution at an excitation wavelength of 488 nm. Histograms of cell cycle were obtained from 3 determinations (100,000 cells/treatment).

### MTS cell viability assay

Preovulatory granulosa cells were transfected with scrambled siRNA and Plk2 siRNA using the Lipofectamine 2000 reagent according to the manufacturer’s instructions. Four hours later, transfection media were replaced with fresh culture media and the cells were treated with hCG (1 IU/ml) for further 24 h. Cell viability was measured using CellTiter 96 AQueous One Solution Cell Proliferation Assay (MTS) according to the manufacturer’s protocol (Promega) as described previously [18]. Briefly, at end of culture, 20 µl of reagent were added into each well and cells were then returned to the incubator for an additional 2 h. The absorbance was measured at 492 nm in the Infinite M200 Pro plate reader (Tecan USA) to determine the formazan concentration, which is proportional to the number of live cells.

### Statistical analyses

All the results are presented as means ± SEM. Two-way ANOVA was used to test differences in Plk2 and Plk3 expression across time of culture and treatment. One-way and t-test analysis of variance (ANOVA) was used to test differences in Plks mRNA levels among treatments. If the effects of time of culture or treatment were revealed significant, the means were compared by Duncan’s test, with *ρ* <0.05 considered significant.
