# Correction: Hormonal Induction of Polo-Like Kinases (Plks) and Impact of Plk2 on Cell Cycle Progression in the Rat Ovary

**DOI:** 10.1371/annotation/1a9779fe-f0ab-4937-a3ce-1bc7fb0268df

**Published:** 2012-08-08

**Authors:** Feixue Li, Misung Jo, Thomas E. Curry, Jing Liu

The following information was missing from the Funding section: The present study was also supported by the National Institutes of Health Grant R01HD057446 to T.E.C. 

