# Correction: Hormonal Induction of Polo-Like Kinases (Plks) and Impact of Plk2 on Cell Cycle Progression in the Rat Ovary

**DOI:** 10.1371/annotation/0e8ec400-2a84-45fe-81d4-bbf20d8124c9

**Published:** 2012-09-07

**Authors:** Feixue Li, Misung Jo, Thomas E. Curry, Jing Liu

There is an error in the Funding statement that was omitted from the first correction. The complete, correct Funding statement is: The present study was supported by the National Natural Science Foundation of China (21007056 to J. L., and 31100845 to F. L.), Scientific Research Foundation of Hangzhou Normal University in China (2011QPL15 to F. L.), and National Institutes of Health Grants (R01HD061617 to M. J., and R01HD057446 to T.E.C.). The funders had no role in study design, data collection and analysis, decision to publish, or preparation of the manuscript. 

